# Psychotherapy and Artificial Intelligence: A Proposal for Alignment

**DOI:** 10.3389/fpsyg.2019.00263

**Published:** 2019-02-11

**Authors:** Flávio Luis de Mello, Sebastião Alves de Souza

**Affiliations:** ^1^Electronic and Computer Engineering Department, Polytechnic School, Federal University of Rio de Janeiro, Rio de Janeiro, Brazil; ^2^Familiacomvida Clinic, São Paulo, Brazil

**Keywords:** brief psychotherapy, degree of differentiation of self, systemic-linking method, cognitive interactive pattern, artificial intelligence

## Abstract

Brief Psychotherapy assists patients to become aware and change their behavior when facing an immediate emotional conflict, and to implement a transformation process through actions of listening, observing, increasing awareness and making interventions. Therapeutic work employs tools and techniques to trigger a process of change, emphasizing cognitive and affective understanding. This article presents an approach that combines Psychology and Artificial Intelligence with the purpose of enhancing psychotherapy with computer-implemented tools. This approach highlights the intersection between these two knowledge areas and shows how machine intelligence can help to characterize affective areas, construct genograms, determine degree of differentiation of self, investigate cognitive interaction patterns, and achieve self-awareness and redefinition. The conceptual proposal was implemented by a web application, and a sample of computer-aided analysis is presented.

## Introduction

This work investigates technological innovation as a tool for the process of psychological recommendation, and addresses the problem of the use of Artificial Intelligence (AI) in the context of Brief Psychological Therapy. Under this perspective, the current work is an effort to point out that processes traditionally regarded as exclusive to the human beings, considered as subjective and complex, can be computed, and that other highly systematic, mechanical and logical processes conceal a certain degree of indeterminism.

There is a worldwide backlash in society, with deconstruction of individual achievements, increased intolerance, emphasis on the narcissistic self, exacerbation of moral and ethical values, and denial of the need for more informed analysis based on scientific knowledge. Such a scenario creates an environment conducive to misguided criticisms of different approaches to problems with traditional solutions that are widely accepted by common sense, and therefore are not very innovative. For this reason, it is a challenge to develop an exploratory study on the nature of the combination of Psychology and Computation.

The term Artificial Intelligence was created by John McCarthy to describe a machine’s ability to perform functions that, if performed by a human being, would be considered intelligent, such as reasoning, learning, decision-making, adaptation, control, and perception. This definition of intelligence for a machine is highly contested because of its pyrotechnic and commercial appeal, and has since been the subject of a very well elaborated critique by McDermott (1981). Regardless of the intentions behind the use of this term, intelligent objects seem to be much desired by the human beings, which makes products with this property eagerly consumed by the public. On the other hand, since intelligence is so desired, there is also a compulsion for exclusive appropriation, giving rise to erroneous comparisons about the possibility of man and machine having the same intelligence, or even the ability to compete. Sometimes, such circumstance is masqueraded as the threat of machine replacing human professionals. This is faulty thinking because “intelligence” itself is not unique or even rare.

Turing (1950) posed the following doubt: “Can machines think?” Such a question introduced the game of imitation, later known as the Turing Test. The proposal disguises the main discussion about the human thought model being unique and exclusive; that is, whether there are other ways of thinking beyond that of the human. The present work agrees with Turing, and assumes that the answer is yes. Therefore, AI not necessarily needs to overtake or overlap human intelligence, but it can be a useful tool to be combined with human intelligence.

Non-human animals have their ways of thinking, certainly distinct from human beings, and yet it makes no sense to compare intelligences with the aim of determining one is superior to another. Thus, if certain entities have their different modes of thinking, then it seems reasonable that machines may also have particular ways of thinking. The important issue is, what is to be done with these different intelligences? And, more specifically, how can an intelligence that is considered “artificial” aid another type of intelligence that is perceived as more “natural”?

Russell and Norvig (2009) provide the most accepted definition for AI today, that is, the designing and building of agents that receive percepts from the environment and take actions that affect that environment. Moreover, the attention AI is currently receiving is very different of the one from the 1990s. At that time, the focus was on logic-based AI, usually under the heading of knowledge representation (KR), whereas today’s focus is on machine learning (ML) and statistical algorithms. The former requires an axiomatic system to perform deductions, and such system is created by retrieving production rules from experts and professionals. The latter requires historical records, usually in a great amount, to create prediction models in order to make inferences (see Mello and Carvalho, 2015). In this article, we argue that a single, or even a medium size group of psychologist, usually do not have such a great quantity of records to enable ML approach. Thus, the method presented in this paper is based on Knowledge Representation.

Regarding this perspective, Psychology can find in Computation support for very specific tasks. Currently, methods that provide support are easily found in Psychology, such as the cathartic method, the free association method and the psychoanalytic research method. This paper proposes that other methods can be adopted, as long as they are in line with what someone wishes to study and develop. For this reason, the fact that an approach is labeled AI does not make it any more, or even less, useful. What is essential is to understand the limits of the approach as a method for assisting someone in problem solving.

The main concern of psychological therapy is to help the individual build self-awareness, that is, to raise its level of awareness about the condition it is going through. In the case of Psychoanalysis, the approach is to listen, observe and raise mindedness of the individual without the implementation of actions of intervention. Brief Psychotherapy, in turn, also listens, observes and raises awareness, but it also performs intervention by providing new information to the patient’s hitherto old views and behaviors. In addition, psychological therapy is also constrained by problems related to the economic use of resources. There are circumstantial inhibitors for many people who seek help: the value of several sessions and the time of travel to the appointment location, for example.

Methods of psychology are used to give referrals about psychological problems, and tools are the resources used by these methods. Such tools, including those implemented by computers, do not contradict the fundamentals of the methods. On the contrary, they aim to support the construction of self-knowledge by the individual, an increase in their discernment, as well as making pertinent information available for the work of the psychotherapist. These same tools are not curative, but relevant assistances for methods with the aim of raising the level of patient self-knowledge, that is, how it perceives and acts in the world. These tools contribute to improving the quality and effectiveness of therapeutic work. Therefore, it is essential to consider tools without classifying them as computational or not, but rather as a resource for therapeutic work, in addition to the other resources that already exist.

An algorithm is understood as a finite set of activities that, when applied in a certain order over a range of data, makes a transformation, producing a certain result. Therapeutic frameworks, systemic interventions, and even analytic approaches, keep in their theoretical foundations rules and spatio-temporal dispositions that refer to this algorithmic methodological aspect. However, although this aspect is necessary, it is not sufficient.

The existence of an algorithm presupposes the control of several variables, their interactions and their effects — something that tends to diverge from the level of predictability of human behavior. The therapist’s clinical insights, the possibilities they identify, and their specific ways of collecting information for later interpretation, provides an approach that allows system process analysis to converge when computational algorithms diverge. Therefore, there is a need for a complement to algorithms that incorporates heuristics. In other words, implemented computational methods need a set of activities capable of conducting deduction that are plausible, but not certain. The processes of Psychology and Computation are not in conflict, but rather are complementary (Gergen, 2016).

Thus, this article explores the results of a hybrid approach that combines Psychology and Artificial Intelligence. The specific objectives are to: (1) study the contribution of machine intelligence tools to the restructuring work of couples, families and individuals in crisis; (2) identify and investigate patterns of functionalities of couples, families, and individuals in crisis through AI techniques and resources; (3) use the Systemic-Linking Method as a mechanism to find the historical bond and the relational/affective patterns of couples, families and individuals; and (4) explore the potential of AI in determining the overall pattern of couples and families by relating it to the specific psychological pattern of the individuals involved.

## Related Work

Psychology uses many tools for data collection and methods for patient evaluation. Murray Bowen devised the genogram as an assessment and intervention tool that provides a graphical representation of family structure, from generation to generation, capable of helping to capture the pattern of interactional functioning of individuals in that family nucleus and its major morbidities. Bowen also created the Differentiation of Self Scale (Bowen, 1991) to understand the degree of emotional maturity of the individual in the context of relational processes. This scale evaluates the individual in contexts where the ego embodies and distinguishes itself from another ego.

Simon (1989) proposed a theory of adaptation from which the quality of subject adjustment is evaluated from four adaptive perspectives (affective-relational, productivity, sociocultural and organic), resulting in the Operational Adaptive Diagnostic Scale. This tool is widely used (Honda and Yoshida, 2012) and has branched into several variations (Yoshida, 2013; Khater, 2014; Peixoto and Yoshida, 2016).

Bertalanffy (1968) created the General System Theory, which points to the integration between the natural and social sciences toward a system theory, where systems are defined as organized modules of elements that are interrelated and that interact among them. Its premise is to look for general value rules that can be applied in any arrangement and at any level of reality. The concept of system is particularly important in Psychology because couples, families, or individuals tend to be understood as systems, sometimes in balance, sometimes far from it. Maturana (1975) created the concept of autopoiesis, which refers to a system being able to self-define, self-construct and, finally, renew itself from these two former actions; a view that also shares concepts with Wiener’s (1954). Cybernetics Theory (Slawomir and Yoshikatsu, 2016). This ability is fundamental to the existence of psychological therapy, since its goal is to lead the couple, family or individual to achieve self-knowledge and discernment, and to move from systemic homeostasis to a new balance in a healthier stable condition. In addition, Prigogine’s (1997). Theory of Dissipative Structures (1997) indicates that disorder (entropy) stimulates the processes of self-organization, and that a system may work both on and off balance, implying a new interpretation of psychopathological phenomena and the psychotherapeutic process.

Freud was one of the pioneers of Brief Psychotherapy, since his early-on work involved treatments that did not usually last more than a year. However, over time, he changed his interest and his studies turned to longer analysis. Ferenczi and Rank (Borgogno, 2001) attempted to introduce changes in the psychoanalytic process in order to reduce time by introducing the term “active technique,” which seeks to make the patient more participative, anticipating their past experiences and propelling them from difficult situations. These authors believed that shortening therapy time was not only just a social and economic matter, but also a technical one. According to them, a predefined number of sessions would induce the patient to stop practicing children’s attitudes toward adult posture.

In Brief Psychotherapy (Knobel, 1986), it is essential to establish extensive knowledge about the patient’s history and personality. Although it may seem time-consuming to collect specific patient data in a context where the number of sessions is limited, the cost is necessary. Deeper knowledge of the patient improves the psychologist work because it accelerates the search for alternative solutions, and thus, shortens the time of therapy.

The first insight into AI combined with psychotherapy is the chatterbot called Eliza (Weizenbaum, 1966), a 1960s natural language processing program created to simulate conversations and give users an illusion of understanding. It is a very important and successful experiment, which was followed by several other bots. However, such software aimed to mimetic a psychologist interacting with a patient, and was never supposed to perform recommendations about patient’s problems. It was during the 1980s that many reports were published describing the support of computer to clinical use (Hartman, 1986; LaChat, 1986; Sampson, 1986; Servan-Schreiber, 1986). These papers proved that logic-based AI could be used as an approach to computerized therapy, particularly to brief cognitive and behavioral therapies. By that time, automatic theorem proving and deduction systems were not mature enough to support such applications, which may be the explanation for the lack of publishing concerning this theme over the next years.

Nowadays, there is a new wave of reports concerning AI and psychotherapy, mainly because the evolution of AI techniques. For instance, Luxton (2014) introduces a computational clinician system concept, which is quite complete. Moreover, there are some initiatives devoted to special issues, such as the one from Morales et al. (2017) who use data mining techniques to distinguish between groups with and without suicide risk. Fitzpatrick et al. (2017) presents a fully automated conversational agent to deliver a self-help program for college students who identify themselves as having symptoms of anxiety and depression. Glomann et al. (2019) describe an application that acts as a constant companion for clinically diagnosed patients who suffer psychological illnesses, supporting them during or after an ambulatory treatment. Besides, there are proposals concerning a wider range of issues. Kravets et al. (2017) presents a full-scale automation of establishing the diagnosis using fuzzy logic for modeling of psychiatrist reasoning. D’Alfonso et al. (2017) discusses the development of the moderated online social therapy web application, which provides an interactive social media-based platform for recovery in mental health.

The process of knowing the patient involves the construction of mental models based on fragmented evidence. The modeling of knowledge is one of the concerns of Artificial Intelligence, since it is necessary to understand human behavior so that a machine can mimic it. Mello and Carvalho (2015) constructed a computational model of representation called Knowledge Geometry, which is agnostic to technology and capable of describing the process of mapping a phenomenon on concepts (intuition) and vice versa (reification). This model also adheres to the process of psychological evaluation in which the professional needs to map the perceived conditions of the patient on the behavioral patterns that belong to Brief Psychotherapy (Yi and Kun, 2017). There is also compliance with the feedback process of designing intervention patterns on the sick patient-system.

When a psychotherapist tries to map and understand the phenomenon that generates a conflict in a patient, there is an attempt to project the theoretical concepts of psychotherapy on the specific situation presented by the individual. The projection of these concepts on the real world is the reification operation of Knowledge Geometry, a process of inference whose resources are analogies and isomorphism. By identifying the modus operandi and the functional pattern of the family (or conjugal system), it becomes possible to intervene and propose new alternatives to the system. Thus, it is possible to deconstruct the addictive mechanisms of feedback and maintenance that prevent the system from admitting new experiences and learnings, thus hampering its development or the resolution of the conflict in question. In AI, this situation is known as Case-Based Reasoning, and is usually modeled by first order logic. On the other hand, when the psychotherapist tries to identify and understand how the patient’s individual symptom is connected to the broader interactional system, that is, how the singular situation is related to the general scenario, it represents the intuition operation of Knowledge Geometry. From this point of view, the patient manifests a symptomatology that is projected onto the family or conjugal system; in other words, the particular phenomenon is used as a support for understanding a broader pattern. In this case, Artificial Intelligence calls a process similar to this Machine Learning.

## Proposal for the Alignment of Psychotherapy and Artificial Intelligence

The process of Brief Psychotherapy involves well-defined steps. Psychological evaluation is the necessary first step for the psychotherapist to know its patient. This phase tends to last around four sessions, depending on the amount of information the patient provides and the diagnostic hypothesis.

The construction of a genogram is performed during this evaluation and can be automated. Moreover, classifications according to the Differentiation of Self Scale and the Operational Adaptive Diagnostic Scale can also be obtained automatically from a pre-established set of questions. Therefore, it is feasible to construct a limited self-service approach capable of generating such information for the psychotherapist.

On the other hand, General System Theory and Cybernetics Theory describe a conceptual system broader than the one used by Computation, and compatible with arrangements in psychology, such as the couple, family and individual. The Theory of Dissipative Structures and the feature of autopoiesis suggest a system capable of defining and renewing itself. All these examples provide evidence that it is possible to explore the potential of the clustering procedure of Artificial Intelligence in predicting the general pattern of couples and families and relate it to the specific pattern of the individual.

The use of Artificial Intelligence can be coupled to the proposed Systemic-Linking Method, which was developed in a school of family psychotherapy and experimented at a social clinic. Such method theoretical bases are connected with General System Theory, Cybernetics Theory, Santiago’s Theory of Cognition (Maturana, 1998) and Bateson’s Theory (Bateson and Ruesch, 2006). Such a method proposes going back to the patient’s past to understand its history and identify unresolved issues (susceptible and vulnerable) that may interfere or enhance the present crisis. Then, the crisis is deconstructed through techniques, exercises and verbal interventions. Finally, patients are encouraged to perform tasks, homework and rituals to introduce new information and insights into the patient-system. Therefore, the system is modified by creating new possibilities for a healthier behavior, which allows the restructuring and rearrangement of the system.

The Systemic-Linking Method presents three distinct stages:

(1) Socio-Historical Contextualizer: this stage seeks to understand and map life experiences (anamnesis) of the couple, family or individual in order to study its historical process in accordance with the transition phases of the family life cycle. During this period, the most relevant task for psychotherapeutic planning is the comprehension of the structural pattern, which is composed of the relational dynamics of the couple, family or individual, the acquisition of familiar historical information, and the genogram.
(2) Integrator: In this second stage, it is necessary to assimilate the patient’s past and present, constrained by the affective bounds they experienced in the family of origin, and which developed the symptomatology in question. Such experiences are updated and re-encountered in the present, and perceived from repetitive emotional patterns. Thus, an investigation of these patterns is carried out, which are reattached and re-enacted through lived and undeveloped experiences that have been frozen in time. The relevant tasks for relational diagnosis are the survey of family mission, the evaluation of its role in the nuclear family, and the Differentiation of Self score concerning the patient’s family.
(3) Interveneer: at this stage, the psychotherapist acts with the patient and their family of origin through exercises, tasks and rituals, in order to reconstruct and re-signify personal bounds, thus creating new meanings for the functioning of the family system. These activities provide new information for the couple, family and individual, promoting the phenomenon of autopoiesis.

## Description of an Experiment

In order to evaluate the usage of AI in the context of Brief Psychotherapy, the present work implemented the Systemic-Linking Method on the computer by employing a deductive artificial intelligence approach based on first-order propositional logic (Robinson, 1965) and fuzzy logic (Shilpa et al., 2016). Such computational implementation is the web-based system called MeetYourself (2018). This section presents two examples of patients with observed conditions and the one obtained by the use of AI.

Each study participant gave informed consent after verbal and written information. All participants signed an informed consent form, their information was anonymized, and only essential information about the participants is reported. The experiment is in accordance to Brazilian Research Ethics Committee (registration number CAAE 90390518.2.0000.5263) and to Declaration of Helsinki. The patients were recruited at the Familiacomvida Psychology Clinic. Subject A is 37 years old, dentist, male, divorced, has two children and is remarried. Its chief complaint concerns depression and marital conflicts. The behavioral observations and mental status examination comprises high degree of anxiety, phobias, fear of dying, pathological rationalization, controlling and centralizing attitude, lack of assertiveness, submissive, aggressive and hostile, dependent on the other’s approval, medium professional autonomy, undeveloped social and entertainment relations, low self-esteem, disorganized and undisciplined. Subject B is 27 years old, vet, female, single, has no children and does not live with its parents. The reasons it is looking for referral are emotional insecurity, fear of ending an old relationship, does not know to say No and is always tries to be a too nice person. The behavioral observations and mental status examination comprises anxiety, controlling attitude, lack of assertiveness, low self-esteem, politically correct, nice person, extremely rational, no professional autonomy, avoid conflicts to avoid destabilize relations, reactive, does not accept being contradicted, angry, verbally aggressive and proud.

A senior psychotherapist developed a set of 140 questions and the possible answers to these questions. Then, for each pair < question, answer > , the psychotherapist created a scored based on their professional judgment of how inadequate each answer was. The intelligence mechanism was constructed to mimic this evaluation, reproducing, in an approximate way, the complex process of the psychotherapist appraising. The answers to these questions comprised exclusive options on a scale from 0 to 4, based on the Likert Scale (Carifio, 2007). Such scale reproduces the level of agreement or disagreement on a symmetrical scale applied over a series of statements.

Although the traditional Systemic-Linking Method is divided into three stages (socio-historical contextualizer, integrator and interveneer), the computational implementation was divided into five large groups of questions because of a systematization strategy. Since the number of questions is so extensive, there is a risk that the patient may withdrawal from the procedure. For this reason, a game dynamics, called gamification (Deterding et al., 2011), was employed to engage the patient and motivate them to continue providing information. The form of awards to encourage the patient is short advice, which is delivered through the system every time a certain number of questions are answered. These are contextualized therapeutic guidelines that can be computationally deducted according to the phase of analysis of the patient.

The first group of questions is related to the 29 queries of the affective area, inspired by Simon’s (1989) Operational Adaptive Diagnostic Scale and its corresponding sectors. In addition to the affective-relational, productivity, sociocultural and organic sectors, a fifth sector, called spiritual, was created, which has the objective of contemplating a relevant dimension that also contributes to the personal and professional satisfaction of the individual (Arcuri and Anconoa-Lopez, 2007). The patient’s responses are scored and classified by a first-order logic inference mechanism supported by a knowledge base. This phase always produces one advice based on the affective-relational sector because it assumes a relevant role of patient behavior, since the relationship precedes the individual (Freud, 1976); that is, every rational system is based on emotion (Maturana, 1998). In addition, a complementary advice is made according to the most recessive sector among the four remaining sectors. The tiebreaker criterion among sectors is Maslow’s Pyramid (Maslow, 1970), with the following ascending order of priority: spiritual, sociocultural, affective-relational, productivity, and organic. Then, the recommendation is produced by a string-manipulation system (Post, 1943) that was implemented for rewriting the propositional logic predicates as natural language, generating a formal language.

**FIGURE 1:**
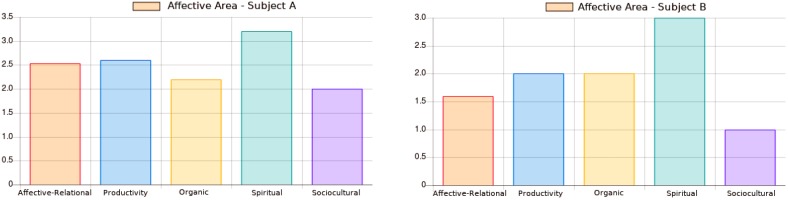
The five affective areas sectors from subjects **(A,B)**. (Translated from Portuguese).

Figure 1 illustrates the graphical output of the five affective areas sectors concerning the answers from subjects A and B. The least developed affective area (lower value), for both subjects, is the sociocultural sector. Thus, at the end of this interaction, both subjects receive recommendations about the affective-relational and sociocultural sector. However, recall that the chronological age of such subjects are not the same. These subjects’ evaluations are mapped into different age ranges, triggering diverse recommendations. Moreover, the answers to the questions were not the same either, which also improves the triggering method by allowing personalized comments. For instance, subject A recommendation includes: *“… Your lack of assertiveness may be interfering on your sociocultural activities. Strengthen the quality of your personal and professional projects by investing and expanding your options of leisure and pleasure. Such expansion can begin with the practice of collective sports.*” Subject B recommendation includes: *“… Your lack of investment in the quality of sociocultural activities may be compromising your personal and professional life. Pay more attention to your social and cultural events as they can become great indicators of how you handle your personal and social life. During such situations, try to listen to different points of view and reflect on them, they may improve your perception.”* The senior psychotherapist prognosis agree with such remarks made by the proposed methodology.

The second group of questions corresponds to 12 queries related to the automated construction of the genogram, which is one of the tools traditionally used by psychotherapists. This group of questions does not produce recommendations for the patient, but rather a descriptive diagram accessible to the psychotherapist. In order to maintain alignment with the concept of gamification, and to keep the patient encouraged through rewards, the implementation of this group of questions is joined by the implementation of a third group of questions, which evaluates the Differentiation of Self score, inspired by the works of Bowen. Bowen defines a scale of 0 to 100, where lower values are associated with one ego very fused to another, while higher values are associated with an ego very different from another.

Despite the common use of Bowen’s scale, this work implements a slightly different and innovative approach to such an analysis. First, it evaluates this differentiation according to age ranges: 0–7, 8–12, 13–19, 20–24, 25–32, and 33 years onward. The expected healthy behavior of a very young child is a cognitive interactional pattern symbiotic to their parents, but does not hold true if the patient is considered an adult person. The method proposed in this paper understands that the chronological age and the emotional age of an individual are relevant during the calculation of the Differentiation of Self score. This temporal characteristic is based on the work of Piaget (2012), in which he associates the organic development of a child with their cognitive development in the sensory-motor, pre-operational, concrete operational and formal operational stages. This requirement of considering the temporal component is also aligned with the works of Freud (1976), who associates the mental functioning pattern of the individual with the oral, anal, phallic, latency and genital phases.

The second important aspect is that Bowen uses a precise deterministic value to measure such differentiation, something that seems to be too rigid to model the human relationship. Thus, in this work, the Differentiation of Self was evaluated in a more flexible way, according to what the author’s call patterns of adaptation, reaction and creativity. The differentiation determination demands an understanding of how an individual interacts with the environment, by building the way they live in the world with some components of autonomy, assertiveness, and self-esteem. Thus, the cognitive interaction pattern is used, so that symptoms analysis is avoided. What is studied is the pattern of mental functioning learned by the individual and that they constructed to survive within a familiar relational context.

According to the pattern of adaptation, the mind tends to accommodate in order to continue in comfort, to preserve pleasure and to avoid pain or suffering, something that is manifested through mechanisms of anxiety and depression. The mind molds itself to the circumstances to avoid effort and energy consumption. The second standard is the pattern of reaction, which concerns a mind that struggles to return itself to wellbeing, while in conflict between leaving the old comfort zone to find a new one, the symptoms of which are aggression or phobias. Finally, the pattern of creativity concerns the individual ability to learn and make solutions possible by creating new possibilities and alternative meanings for situations of displeasure, pain, suffering and discomfort. The patient’s perception is amplified when facing difficulties, and thus the new information is more easily assimilated.

The change between interactional patterns, that is, the migration from one pattern to another, allows the patient to improve their way of perceiving and acting in the world. Therefore, the need to evaluate the Differentiation of Self requires considering the three patterns simultaneously, and not exclusively, which is incompatible with the original deterministic method proposed by Bowen.

Artificial Intelligence often makes use of Fuzzy Logic (Shilpa et al., 2016) to process problems with imprecise variables, which is an interesting approach for modeling and computing a preliminary patient mental status examination based on the Differentiation of Self. Hence, 17 queries were constructed related to the pattern of adaptation, 21 queries on the pattern of reaction and 19 queries on the pattern of creativity. The responses feed the Zadeh (1965) evaluation model and the results reflect three degrees (adaptation, reaction and creativity) of pertinence to the cognitive interactional pattern. Thus, these degrees of Differentiation of Self allow the AI to produce recommendations based on the chronological/emotional age of the patient and on the pertinence to the individual’s cognitive interactional pattern.

**FIGURE 2:**
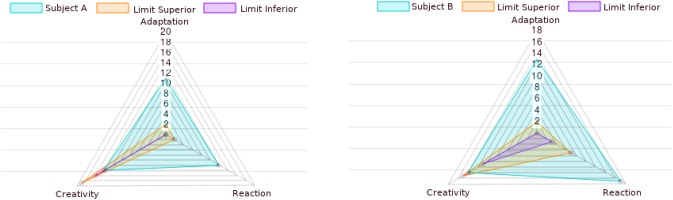
Radar charts of cognitive interaction patterns for subjects **(A,B)**, based on degrees of differentiation of self.

Figure 2 shows the system graphical output of the cognitive interaction patterns based on degrees of differentiation of self. At these radar charts, there are limits superior and inferior for such patterns (adaptation, reaction and creativity). The healthy evaluations indicates subjects’ cognitive interaction patterns inside these intervals. Moreover, observe that such limits depends on the chronological age from the subjects. Subjects A and B are both inadequate concerning adaptation and reaction. Subject A has a high value for adaptation and this is compatible with the symptoms observed by its psychotherapist: panic disorder, low self-esteem and tolerant with the conjugal infidelity of its spouse. The psychotherapist also observed that the high value for reaction is suited for someone, as subject A, with phobias, and whose behavior is aggressive and hostile. The stereotype given by the professional to this subject is of an adolescent. Subject B, on its turns, has also a high value of adaptation, and according to its psychotherapist, this is consistent with its symptoms: the symbiotic relationship with its mother and the complete financial dependency of its parents. Moreover, its reaction score is compatible with its difficulty to accept being contradicted, angry and verbally aggressive behavior, and proud. The stereotype given by its psychotherapist is of a spoiled little person.

The fourth group of questions concerns awareness and redefinition, and is composed of 33 queries. The patient is led to reflect on the subject of the questions, making them aware of unconscious defense mechanisms that interfere with good resolution to problems. The AI system uses the patient’s responses to quantify emotional maturity in relation to their social cycle. An inference mechanism, the core of an Artificial Intelligence engine called automatic theorem prover, allows this classification, and then recommendations are made according to the inferred class for the patient.

Subjects A and B answers to the questions from this third group activated recommendation about assertiveness, self-esteem and autonomy. The therapist also considered three aspects relevant. Since this stage of the method aims to improve patient awareness and redefinition, the system also needs to trigger mechanisms concerning the specific features of subject disequilibrium. The implemented method successfully identified the need for providing to subject A comments on relationship, and to subject B comments on differentiation. This then ends the Integrator stage.

The final stage of the Systemic-Linking Method is the Interveneer stage, which aims to provide change to the patient’s affective, cognitive and behavioral domains. The prototype implementation described in this paper contains nine queries that have the function of collecting more information for the psychotherapist, and of indicating intervention exercises. These exercises are based on the mainstream science of family psychotherapy and on the AI clustering technique. Such exercises work as an instrument to manage patient resistance in relation to the conflict that needs a referral. They promote patient compromise on treatment, inducing them to perform transformation actions in real life, outside the psychotherapeutic office.

Each item evaluated throughout the process of assessing the patient’s mental behavior pattern has a corresponding exercise (affective area, pattern of adaptation, pattern of reaction, pattern of creativity, relationship, Differentiation of Self, autonomy, assertiveness, self-esteem). The choice of exercises to be assigned to the patient is based on a classificatory system implemented with first order logic, which uses a knowledge base to infer the relevance of each activity. Consequently, the patient receives a kind of customized homework to be practiced and delivered to their psychoanalyst.

The hybrid approach of using Psychology and Artificial Intelligence in order to assist therapists by providing computer-implemented tools, as proposed in the present paper, allows combining heuristics and algorithms in a complementary way. All patient answers, evaluations and recommendation are available to the psychotherapist. The approach does not involve a conflictual relationship between these research fields, but a potentially complementary one. AI can act as tool for psychotherapy, as many other existing tools do. In addition, this combination enables a preliminary self-care approach capable of generating information for the psychotherapist and a therapeutic orientation for the patient. The former is able to anticipate certain understandings about the patient, while the latter receives useful guidance for improving self-knowledge and self-understanding.

Therefore, the proposed method reduces the need for in-person sessions, but it do not eliminate all of them. The experiments indicate that, when considering a Brief Psychotherapy model of ten (10) sessions, the system can be an alternative for up to the first four (4) sessions of therapy, automating a great portion of the anamnesis work. It delivers compiled information to the psychotherapist such as genogram, social historical data, differentiation of self score, cognitive interaction pattern and mental behavior pattern. The patient receives a set of exercises that promote changes at its perception, and this new perception encourages the remodeling of emotions. When such exercises are taking action the patient begins to change its behavior.

Although the method improves the therapeutic work, it is not curative, it just assists patient and psychologist. Thus, there are limits to this approach as a method for assisting someone in problem solving. The therapist can, and a computational method cannot, work with high-level abstractions, analogies and metaphors. A great challenge is to perform the association between patients past and its present, allowing a succession of decontextualization and recontextualization actions. The proposed method is far beyond being able to perform the reconstruction of thinking, that is, to take a line of thought and patients discourse, carry out a synthesis and create something new: the positive connotation. The therapist can reframe certain situations into a new perception field. This method, on its turn, cannot do it, even in simple circumstances. For instance: a patient complains that it mother is too angry and intolerant; the mother has five (5) children, no husband or family support; the computer cannot formulate a hypothesis of this mother being too angry or being protective.

## Conclusion

The present essay addresses the feasibility of combining Psychology and Artificial Intelligence; in other words, how Psychology can find support for specific tasks in Computation. An Artificial Intelligence approach does not make the computational support more or less useful, and the limits of such an approach as a method for solving a given problem must be understood. From this perspective, AI can play a role as an add-on resource for therapeutic work, in addition to those that already exist.

The Systemic-Linking Method is a suitable alternative for implementing computational intelligence as an auxiliary tool in determining the behavioral pattern of couples, families and individuals. Among the several approaches of Artificial Intelligence, first order logic, automatic theorem prover, and fuzzy logic can be used to implement the procedures related to the characterization of affective areas, genogram construction, Differentiation of Self determination, cognitive interaction pattern evaluation, improvement of self-awareness and redefinition, to finally collaborate with psychological interventions.

This study proposes that the activity of overlaying the functional pattern of a family system over the phenomena that generates conflict for the individual resembles the reification procedure of Knowledge Geometry theory (Mello and Carvalho, 2015). Artificial Intelligence models address such circumstances by methods of knowledge-based reasoning. Furthermore, when a patient’s symptomatology is projected onto the family or conjugal system, there is something similar to the computational procedure of intuition, otherwise known as machine learning.

The immediate future work is to integrate the separate implementations constructed for the Systemic-Linking Method into a single system. It is expected that an integrated system can better promote a brief therapeutic orientation, and also provide a service to attract patients to professional psychotherapists. Additional future work involves comparing the evaluations and recommendations produced by the system with those made by a psychotherapist. This type of blind test will help measure the effectiveness of the system, as well as contribute to any necessary fine-tuning. Finally, continued investigation into combining Psychology and Artificial Intelligence is recommended, particularly with regard to exploring the concepts and mechanisms that trigger the behavioral patterns of an individual within the taxonomy of Marvin Minsky’s frames (Minsky, 1987). It may also be interesting to investigate the usage of Machine Learning on Psychotherapy.

## Author Contributions

All authors listed have made a substantial, direct and intellectual contribution to the work, and approved it for publication.

## Conflict of Interest Statement

The authors declare that the research was conducted in the absence of any commercial or financial relationships that could be construed as a potential conflict of interest.
